# Resonance-Assisted Depinning of DMI-Stabilized Néel Domain Walls in Stepped Perpendicular Magnetic Nanowires Driven by Pulsed Currents

**DOI:** 10.3390/nano16181136

**Published:** 2026-09-10

**Authors:** Mohammed Al Bahri, Salim Al-Kamiyani, Eduardo Saavedra, David Laroze, Felipe Tejo

**Affiliations:** 1 Department of Basic and Applied Sciences, A’Sharqiyah University, P.O. Box 42, Ibra 400, Oman; 2 Department of Physics, University of Santiago de Chile, Av. Victor Jara 3493, Santiago 9170124, Metropolitan Region, Chile; 3 Instituto de Alta Investigación, Universidad de Tarapacá, Arica 1000000, Chile; 4 Vicerrectoría de Investigación, Innovación y Postgrado, Universidad Central de Chile, Toesca 1783, Santiago 8370178, Metropolitan Region, Chile

**Keywords:** domain wall dynamics, domain wall depinning, perpendicular magnetic anisotropy, Dzyaloshinskii–Moriya interaction, Néel domain wall, spin-transfer torque, pulsed current excitation, resonance-assisted depinning, stepped pinning sites, spintronic memory devices

## Abstract

Current-driven manipulation of magnetic domain walls (DWs) in perpendicularly magnetized nanowires is promising for spintronic memory and logic applications. In this work, micromagnetic simulations are used to investigate the depinning of DMI-stabilized Néel domain walls from a stepped pinning site under unipolar square-pulse current excitation. The effects of current density, pulse frequency, duty cycle, Dzyaloshinskii–Moriya interaction (DMI), and temperature are examined. The results show that pulse frequency and duty cycle strongly influence the oscillatory response of the pinned DW and can promote depinning through efficient coupling with localized DW dynamics. Increasing the current density enhances the oscillation amplitude and facilitates escape from the pinning region, while variations in DMI produce marked changes in pinning stability and magnetic configuration. Thermal fluctuations further modify the depinning behavior, particularly for stronger DMI, where the system evolves from stable pinning to thermally assisted escape and, at higher temperatures, to less stable magnetic states. Overall, the results show that pulse parameters, DMI strength, and temperature jointly control DW depinning and stability, providing further insight into the tuning of current-driven DW transport in geometrically confined spintronic structures.

## 1. Introduction

Magnetic domain walls (DWs) have attracted considerable attention owing to their potential applications in next-generation spintronic devices, including racetrack memories, magnetic logic circuits, and neuromorphic computing systems [1,2,3]. In these devices, information is stored in magnetic domains and manipulated through controlled DW motion driven by magnetic fields or spin-polarized currents [4,5,6]. Compared with conventional semiconductor technologies, DW-based devices offer several advantages, including non-volatility, high storage density, low power consumption, and fast switching speeds [1,2].

Perpendicularly magnetized nanowires (PMNs) have emerged as promising candidates for DW-based memory applications because perpendicular magnetic anisotropy (PMA) provides enhanced thermal stability and allows the formation of narrow domain walls suitable for high-density information storage [7,8,9,10]. However, the reliable manipulation of DWs remains a major challenge because their propagation is strongly influenced by intrinsic and extrinsic pinning sites, which can hinder motion and increase the energy required for depinning [11,12,13].

To achieve deterministic control of DW motion, geometrical modifications such as notches, constrictions, antidots, and stepped regions have been widely employed to create controllable pinning sites within magnetic nanowires [14,15,16,17,18]. These engineered defects can act as stable storage locations for DWs and therefore play a crucial role in the operation of racetrack memory devices. Consequently, understanding the interaction between DWs and geometrical pinning potentials is essential for the development of reliable spintronic technologies [16,17,18].

Recent studies have demonstrated that spin-polarized currents can efficiently drive DW motion through spin-transfer torque (STT) mechanisms [19,20,21,22]. The depinning behavior depends strongly on the current density, pulse duration, material properties, and nanowire geometry [20,21,22,23,24]. Although extensive investigations have been performed on current-driven DW depinning, most studies have focused on continuous current excitation [23,24,25,26]. In practical devices, however, pulsed-current operation is preferred because it can significantly reduce Joule heating and energy consumption while providing additional flexibility for controlling DW dynamics [27,28,29,30].

Pulsed-current excitation introduces several parameters that can strongly influence DW behavior, including pulse frequency, pulse width, and duty cycle [27,28,29,30,31]. While the effects of current density and pulse duration have been widely explored, the influence of duty cycle on resonance-assisted DW depinning remains poorly understood. In particular, the existence of critical duty-cycle thresholds capable of triggering transitions between pinned, oscillatory, and depinned states has received limited attention in stepped PMA nanowires [30,31].

Another important factor governing DW dynamics is the Dzyaloshinskii–Moriya interaction (DMI), which originates from strong spin–orbit coupling in systems lacking inversion symmetry [32,33,34,35]. DMI stabilizes chiral Néel domain walls and significantly modifies their static and dynamic properties, including DW velocity, chirality, stability, and depinning behavior [33,34,35,36,37,38]. Furthermore, strong DMI may lead to the formation of complex chiral magnetic textures such as skyrmions and spin spirals [35,36,37,38,39]. Despite substantial progress in understanding DMI-driven DW dynamics, the combined influence of DMI and pulsed-current excitation on resonance-assisted depinning in geometrically confined PMA nanowires has received comparatively limited attention [37,38,39,40].

Temperature also plays a significant role in DW motion because thermal fluctuations can alter the effective pinning potential and modify the stability of magnetic configurations [41,42,43,44]. Previous studies have shown that thermal activation can either facilitate or suppress depinning depending on the magnetic properties and geometrical characteristics of the nanowire [42,43,44,45]. Nevertheless, the interplay among temperature, DMI, and pulsed-current excitation in stepped PMA nanowires remains largely unexplored.

Motivated by these considerations, the present work investigates the resonance-assisted dynamics and depinning behavior of DMI-stabilized Néel domain walls in a stepped PMA nanowire driven by unipolar square pulsed currents. The effects of current density, pulse frequency, duty cycle, DMI strength, and temperature are systematically examined. Dynamical phase diagrams are constructed to identify transitions between pinned oscillatory states, nonlinear oscillatory regimes, and complete depinning. Furthermore, the influence of DMI on oscillation amplitude, domain wall configuration, and thermal stability is analyzed. The obtained results provide new insights into the control of DW motion through pulse engineering and chiral interactions in stepped spintronic structures.

Previous studies have investigated resonant domain-wall excitation and depinning under time-dependent current excitation, as well as the influence of DMI on domain-wall dynamics. In the present work, we focus on the resonance-assisted dynamics and depinning of DMI-stabilized Néel domain walls in a stepped PMA nanowire under unipolar square-pulse current excitation. The study systematically examines how current density, pulse frequency, duty cycle, and DMI strength modify the dynamical response of the pinned DW, including transitions between pinned oscillation, nonlinear oscillation, deformed DW configurations, extended chiral textures, spin-spiral-like states, and complete depinning. We further examine the robustness of these dynamical behaviors against thermal fluctuations. This approach provides insight into how pulse characteristics and chiral interactions jointly control the dynamical pathways leading to DW depinning in a geometrically confined system.

## 2. Theoretical Model and Simulation Methodology

**Figure 1:**
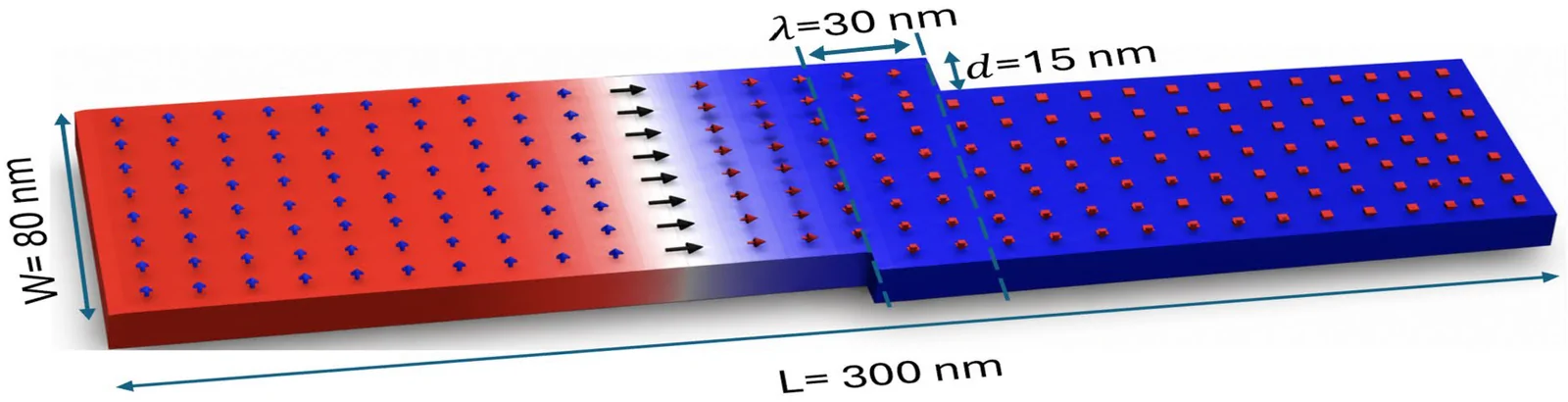
Three-dimensional schematic of the stepped perpendicularly magnetized nanowire showing the geometrical dimensions used in the simulations (L = 300 nm, W = 80 nm, λ = 30 nm, d = 15 nm).

The dynamic behavior of a Dzyaloshinskii–Moriya interaction (DMI)-stabilized Néel domain wall (DW) in a stepped perpendicularly magnetized nanowire was investigated using the MuMax3 micromagnetic simulation package. The simulated nanowire consists of a rectangular strip with dimensions L × W × t, where L, W, and t represent the nanowire length, width, and thickness, respectively, as shown in Figure 1. Preliminary simulations indicated that smaller step depths produced weak pinning, whereas larger depths led to strong trapping and suppressed domain-wall motion. Therefore, a step depth of d=15  nm and a width of λ=30  nm were chosen as representative dimensions that place the system near the pinning–depinning threshold, allowing systematic investigation of frequency, duty-cycle, DMI, and temperature-dependent dynamics.

A stepped geometrical defect was introduced to act as a localized pinning site for the DW. The interplay among spin-transfer torque, DMI, thermal fluctuations, and geometrical pinning governs the DW dynamics under unipolar square pulsed-current excitation. The geometrical dimensions, magnetic properties, and simulation parameters used in this work are summarized in Table 1.

The temporal evolution of the normalized magnetization vector, $m=M/M_{s}$, was described by the Landau–Lifshitz–Gilbert (LLG) equation including spin-transfer torque contributions [46,47,48,49]:

(1) $$\frac{\partial m}{\partial t}=-\gamma m\times H_{eff}+\alpha m\times \frac{\partial m}{\partial t}+\left(u\cdot \nabla \right)m-\beta \;m\times \left[\left(u\cdot \nabla \right)m\right]$$

(2) $$u=\frac{P\mu_{B}}{eM_{s}\left(1+\beta^{2}\right)}J$$

Here, γ is the gyromagnetic ratio, α is the Gilbert damping constant, and H_eff_ is the effective magnetic field. The last two terms describe the adiabatic and non-adiabatic components of the Zhang–Li spin-transfer torque, respectively [21]. The vector u represents the spin-drift velocity, where P is the spin polarization, μ_B_ is the Bohr magneton, e is the elementary charge, M_s_ is the saturation magnetization, and **J** is the applied current-density vector. In MuMax3, the non-adiabaticity parameter β is specified by the parameter ξ. In the present simulations, P = 0.4 and β = ξ = 0.1 were used. The effective magnetic field was obtained from the variational derivative of the total magnetic energy density [50]:

(3) $$H_{eff}=-\frac{1}{\mu_{0}M_{s}}\frac{\delta E}{\delta m}$$

where $\mu_{0}$ is the vacuum permeability and E is the total magnetic energy density.

The total magnetic energy consists of exchange, anisotropy, demagnetization, Zeeman, and DMI contributions [51,52]:

(4) $$E=E_{exch}+E_{anis}+E_{demag}+E_{Zeeman}+E_{DMI}$$

The exchange energy density is given by [50]

(5) $$E_{exch}=A\left[(\nabla m_{x}{)}^{2}+(\nabla m_{y}{)}^{2}+(\nabla m_{z}{)}^{2}\right]$$

where A is the exchange stiffness constant.

The perpendicular magnetic anisotropy energy density is expressed as [53]

(6) $$E_{anis}=-K_{u}m_{z}^{2}$$

where $K_{u}$ is the perpendicular magnetic anisotropy constant.

The interfacial Dzyaloshinskii–Moriya interaction energy density is written as [32,33,54]:

(7) $$E_{DMI}=D\left[m_{z}(\nabla \cdot m)-(m\cdot \nabla )m_{z}\right]$$

where D is the DMI constant. This term stabilizes chiral Néel domain walls and strongly influences the wall structure, pinning behavior, oscillation dynamics, and depinning efficiency.

Current-induced DW motion was generated using a unipolar square pulsed current applied along the nanowire axis. The pulse frequency is defined as [55]:

(8) $$f=\frac{1}{T_{p}}$$

where $T_{p}$ is the pulse period.

The duty cycle is defined as

(9) $$DC=\frac{t_{on}}{T_{p}}$$

where $t_{on}$ is the pulse ON duration. Increasing the duty cycle increases the effective duration of spin-transfer torque acting on the DW during each pulse cycle.

Thermal fluctuations were incorporated using Brown’s thermal field model. The thermal field has a zero average value [56]:

(10) $$\langle H_{th,i}(t)\rangle =0$$

and satisfies the correlation relation

(11) $$\left\langle H_{th,i}(t)H_{th,j}(t^{\prime })\rangle =\frac{2\alpha k_{B}T}{\gamma \mu_{0}M_{s}V}\delta_{ij}\delta (t-t^{\prime }\right)$$

where $k_{B}$ is the Boltzmann constant, T is the temperature, V is the computational cell volume, and δ  is the Dirac delta function.

For the finite-temperature analysis, five statistically independent simulations were performed at each temperature using different thermal-noise seeds. The mean values and corresponding standard deviations were calculated from these independent realizations. The step-escape probability was defined as P_esc_ = N_esc_/N, where N_esc_ is the number of trajectories in which the DW crossed the stepped pinning region and N = 5 is the total number of independent realizations. For conditions in which not all trajectories escaped, the mean step-escape time was calculated only from the trajectories that exhibited an escape event. This statistical treatment was used to quantify the trajectory-to-trajectory variability introduced by thermal fluctuations.

The DW oscillation amplitude was quantified from the temporal evolution of the transverse magnetization component $m_{y}$ within the stepped region and calculated as [57]:

(12) $$A_{osc}=\frac{m_{y,max}-m_{y,min}}{2}$$

where $m_{y,max}$ and $m_{y,min}$ are the maximum and minimum values of $m_{y}$, respectively. Although the current excitation is unipolar, the oscillation amplitude is conventionally defined as half of the peak-to-peak variation in the measured magnetization signal. Since $m_{y}$ is the normalized transverse magnetization component, the oscillation amplitude $A_{osc}\;$ is dimensionless.

For the spectral analysis, the local transverse magnetization signal m_y(t) near the stepped region was analyzed over a common time window from 2 to 18 ns. The signal was sampled at 5 ps intervals. Before applying the FFT, the mean value was removed, and a Hann window was applied to reduce spectral leakage. For the two-dimensional spectral map, the FFT amplitudes were normalized with respect to the maximum spectral amplitude over the analyzed excitation-frequency range.

Resonance-assisted depinning occurs when the periodic current excitation efficiently couples to the characteristic oscillatory dynamics of the pinned domain wall in the considered geometrical configuration. The spin-transfer torque acts as a periodic excitation of the DW, inducing localized oscillatory motion around the stepped pinning region. When the excitation is efficiently synchronized with this dynamical response, the oscillation amplitude is enhanced over successive pulse cycles. The resulting large-amplitude DW motion facilitates the crossing of the geometrical pinning barrier, ultimately leading to depinning.

A domain wall was considered depinned when it permanently escaped from the stepped region and propagated toward the end of the nanowire. Conversely, configurations in which the DW remained trapped or oscillated around the stepped region throughout the simulation were classified as pinned states. By systematically varying the current density, pulse frequency, duty cycle, DMI strength, and temperature, the transitions between pinned oscillation, nonlinear oscillation, deformed DW states, extended chiral textures, spin-spiral-like configurations, and complete depinning were investigated and summarized through dynamical phase diagrams. All simulations were initialized from a relaxed Néel-type domain wall located before the stepped pinning region. The initial magnetic configuration was first relaxed at zero current before applying the pulsed-current excitation. The stepped region had a width of λ=30  nm and a depth of d=15  nm and was introduced as a localized geometrical pinning site in the nanowire. Since the present study considers a single localized geometrical pinning site, the frequency-dependent depinning behavior discussed below refers specifically to the dynamical response of the DW in this particular pinning configuration. Different pinning geometries or multiple pinning sites may exhibit different characteristic oscillatory responses. Unless otherwise stated, open boundary conditions were used along the nanowire directions. For the frequency-dependent simulations, a zero-current relaxation was first performed for 2 ns, followed by pulsed-current excitation for 100 ns. Additional zero-current relaxation was then applied to confirm whether the DW remained pinned or had permanently depinned. A DW was classified as depinned when it escaped from the stepped region and propagated toward the end of the nanowire without returning to the pinning site. Conversely, if the DW remained trapped near the stepped region or exhibited localized oscillatory motion throughout the simulation time, it was classified as pinned. The unipolar square-pulse current was implemented by applying a current density J during the ON interval and setting the current to zero during the OFF interval. For the limiting case of duty cycle DC=1, the current remained continuously ON, corresponding to continuous-current excitation.

## 3. Results and Discussion

**Figure 2:**
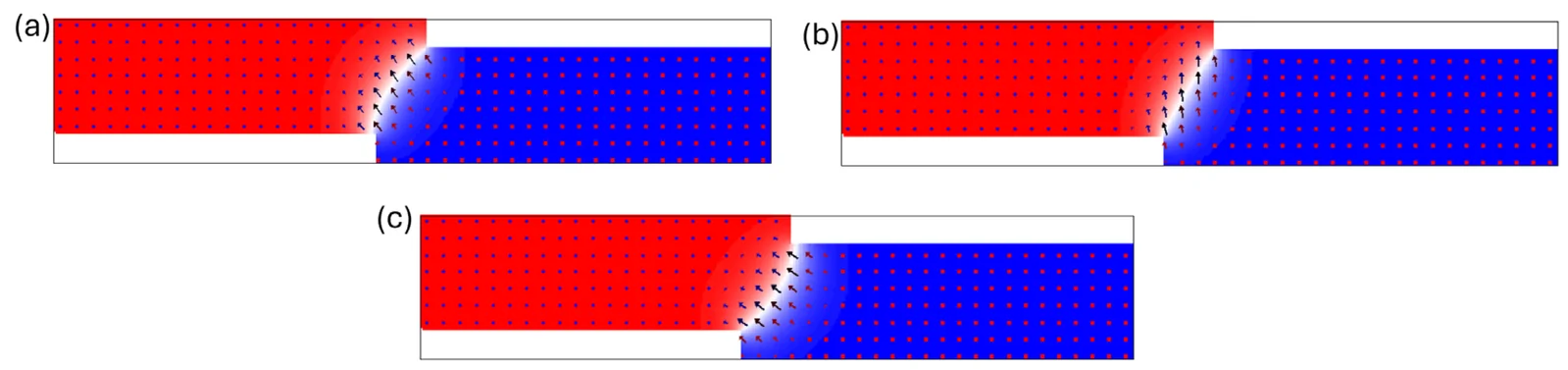
Representative micromagnetic snapshots of the DMI-stabilized Néel domain wall (DW) interacting with the stepped pinning region under unipolar square-pulse current excitation. (**a**) Initial interaction with the stepped barrier, (**b**) tilted and deformed DW during localized oscillation, and (**c**) enhanced deformation and displacement near the pinning site. The simulations were performed at T = 0 K.

Figure 2 presents representative micromagnetic snapshots of the DMI-stabilized Néel domain wall (DW) interacting with the stepped pinning region under unipolar square-pulse current excitation. The sequence illustrates the transient evolution of the DW during its localized oscillatory motion near the geometrical barrier. In Figure 2a, the DW approaches the stepped region and begins to experience deformation due to the interaction between the pulse-driven spin-transfer torque and the local pinning potential. In Figure 2b, the DW undergoes pronounced tilting and internal spin reorientation while remaining confined near the step, indicating strong excitation of its internal degrees of freedom. In Figure 2c, the DW is displaced further within the vicinity of the stepped region and exhibits enhanced deformation, reflecting the nonlinear character of the oscillatory dynamics.

The snapshots demonstrate that the DW does not behave as a rigid magnetic interface. Instead, its width, inclination angle, and internal spin configuration continuously evolve during the oscillation process. The localized deformation observed near the stepped region indicates the presence of a restoring force generated by the geometrical discontinuity. Under the action of the pulsed spin-transfer torque, the DW is periodically displaced from its equilibrium position, whereas the stepped barrier tends to restore it toward a lower-energy pinned configuration. The competition between pulse-driven excitation and geometrical confinement gives rise to the observed localized oscillatory behavior.

Furthermore, the figure reveals significant transient modifications of the Néel-wall structure during the oscillation cycle. The increasing deformation and displacement observed from Figure 2a–c indicate efficient energy transfer from the pulsed current into the DW internal modes. Such behavior becomes particularly important near resonance-assisted depinning conditions, where enhanced oscillation amplitudes may facilitate eventual depinning. Therefore, Figure 2 provides direct micromagnetic evidence that the observed dynamics originate from the combined effects of DMI stabilization, pulse-induced spin-transfer torque, and geometrical confinement at the stepped pinning site.

**Figure 3:**
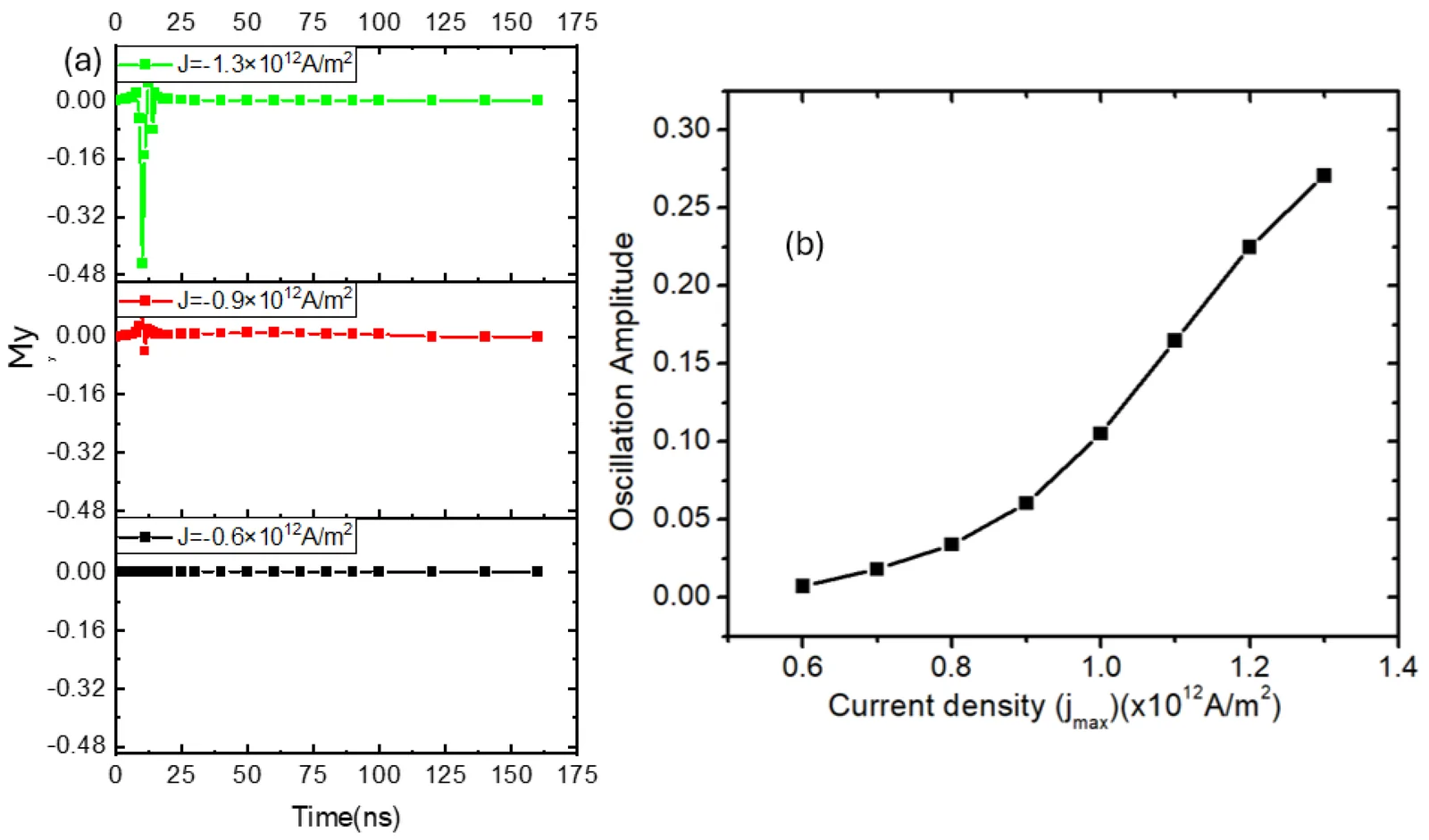
(**a**) Temporal evolution of the local transverse magnetization component $m_{y}$ for different current densities. (**b**) Oscillation amplitude $A_{osc}$ as a function of current density. The results illustrate the effect of current density on the dynamics of the DMI-stabilized domain wall near the stepped pinning region. The simulations were performed at T = 0 K.

Figure 3a presents the temporal evolution of the local transverse magnetization component $m_{y}$ measured near the stepped pinning region under different unipolar square-pulse current densities. At the lower current density of $J=0.6\times 10^{12}\;A/m^{2}$, only weak oscillatory behavior is observed, indicating that the injected spin-transfer torque is insufficient to strongly perturb the pinned DMI-stabilized Néel domain wall (DW). As the current density increases to $0.9\times 10^{12}\;A/m^{2}$, the oscillation amplitude becomes noticeably larger, demonstrating enhanced localized DW excitation while the stepped geometry still effectively confines the wall. A further increase to $1.3\times 10^{12}\;A/m^{2}$ produces a dramatic amplification of the oscillation amplitude accompanied by strong nonlinear transient dynamics prior to complete depinning. The sharp negative and positive excursions in $m_{y}$ indicate intense internal DW deformation and instability immediately before the DW escapes the stepped region and propagates toward the end of the nanowire. These results confirm the existence of a current-driven transition from weak localized oscillation to nonlinear oscillatory dynamics and eventual depinning under square-pulse excitation.

Figure 3b summarizes the dependence of the oscillation amplitude on the applied current density. The oscillation amplitude increases monotonically with increasing current density, revealing progressive enhancement of the localized DW dynamics under stronger spin-transfer torque. At lower current densities ($0.6-0.8\times 10^{12}\;A/m^{2}$), the oscillation amplitude remains relatively small, indicating stable DW confinement by the stepped pinning potential. However, beyond $0.9\times 10^{12}\;A/m^{2}$, the oscillation amplitude rises rapidly, suggesting the onset of nonlinear DW dynamics and increasing competition between the spin-transfer torque and the geometrical restoring force of the stepped region. Near the threshold current density, the strong enhancement in oscillation amplitude acts as a precursor to depinning. At $1.3\times 10^{12}\;A/m^{2}$, the oscillation amplitude reaches its maximum value, corresponding to the transition from localized oscillatory confinement to complete DW depinning. Overall, the figure demonstrates that unipolar square-pulse current excitation enables efficient control of localized DW oscillation and the pinning-to-depinning transition in stepped PMA nanowires.

**Figure 4:**
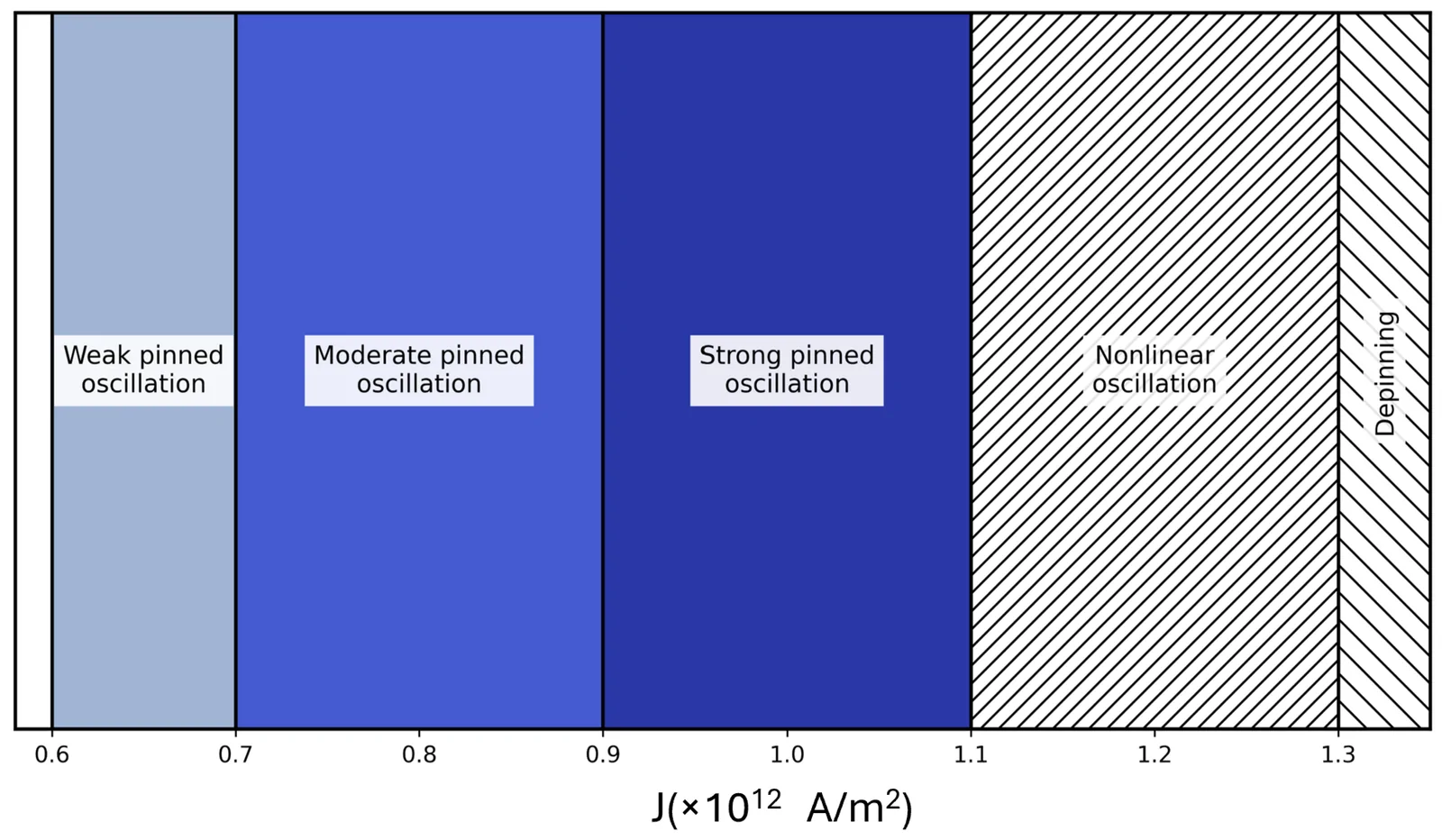
Current-density-dependent dynamical phase diagram of the DMI-stabilized Néel domain wall (DW) under unipolar square-pulse current excitation. The colored and hatched regions identify weak, moderate, and strong pinned oscillations, nonlinear oscillation, and complete depinning as functions of the applied current density. The simulations were performed at T = 0 K.

Figure 4 presents the current-density-dependent dynamical phase diagram of the DMI-stabilized Néel domain wall (DW) in the stepped PMA nanowire under unipolar square-pulse current excitation. The phase diagram summarizes the evolution of the DW dynamics as the applied current density increases and identifies five distinct dynamical regimes: weak pinned oscillation, moderate pinned oscillation, strong pinned oscillation, nonlinear oscillation, and complete depinning.

At low current densities (J = 0.6 × 10^12^ A/m^2^), the DW remains strongly confined at the stepped pinning site and exhibits only weak localized oscillations. In this regime, the spin-transfer torque is insufficient to significantly perturb the equilibrium wall position, and the geometrical restoring force dominates the dynamics.

Increasing the current density to J = 0.7–0.8 × 10^12^ A/m^2^ produces moderate pinned oscillations. Although the DW remains trapped by the stepped barrier, the larger oscillation amplitude indicates more efficient energy transfer from the pulsed current into the DW internal modes.

A further increase to J = 0.9–1.0 × 10^12^ A/m^2^ results in strong pinned oscillatory behavior. The enhanced oscillation amplitude reflects increased competition between spin-transfer torque and the geometrical pinning potential. However, the injected energy remains insufficient for complete barrier crossing, and the DW continues to oscillate around the stepped region.

Near the critical current-density regime (J = 1.1–1.2 × 10^12^ A/m^2^), the system enters a nonlinear oscillation state characterized by large-amplitude transient motion and pronounced DW deformation. This regime represents an intermediate dynamical state between stable pinning and complete depinning, where the injected energy becomes comparable to the effective pinning barrier.

The progressive increase in oscillation strength with current density suggests continuous energy accumulation within the pinned domain-wall mode. As the spin-transfer torque increases, larger amounts of energy are transferred to the domain wall during each pulse cycle, resulting in enhanced oscillation amplitudes and stronger internal deformation. The nonlinear oscillation regime observed near $J=1.1–1.2\times 10^{12}$ A/m^2^ represents a precursor state to depinning, where the injected spin-transfer torque becomes comparable to the effective pinning potential of the stepped barrier. In this regime, the domain wall repeatedly approaches the depinning threshold but remains temporarily confined by the geometrical restoring force, indicating the onset of resonance-assisted barrier crossing.

At J = 1.3 × 10^12^ A/m^2^, complete depinning occurs and the DW escapes from the stepped region, propagating toward the end of the nanowire. This transition indicates that the spin-transfer torque has exceeded the effective pinning strength of the geometrical barrier.

Overall, the phase diagram reveals a progressive current-driven transition from weak pinned oscillation to nonlinear excitation and eventual depinning. The results demonstrate that the applied current density serves as an effective control parameter governing energy accumulation, oscillation amplification, and depinning of DMI-stabilized domain walls in stepped PMA nanowires.

**Figure 5:**
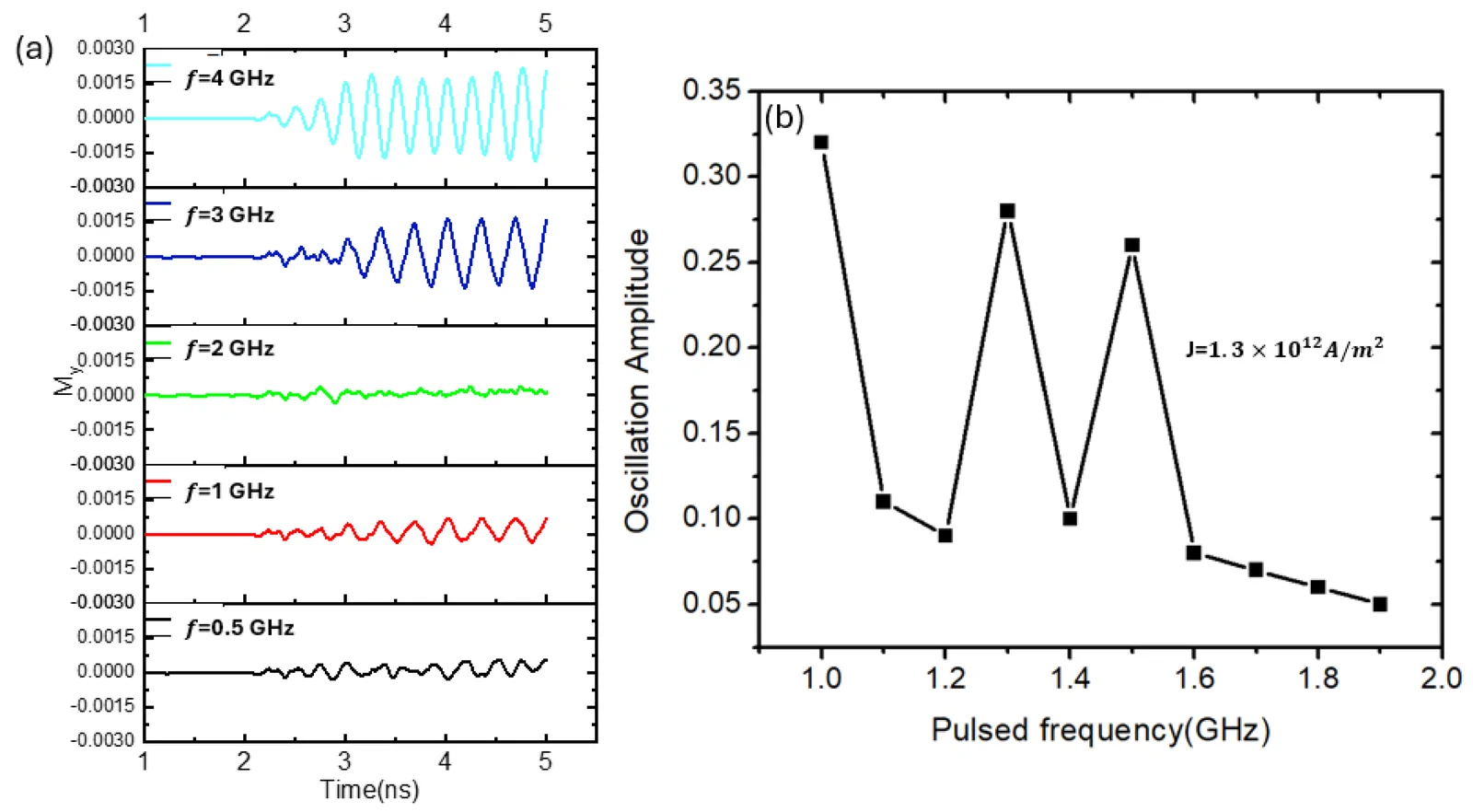
(**a**) Temporal evolution of the local transverse magnetization component $m_{y}$ near the stepped pinning region under different pulse frequencies at a fixed current density of $J=0.9\times 10^{12}\;A/m^{2}$. (**b**) Oscillation amplitude, $A_{osc}$, as a function of pulse frequency at $J=1.3\times 10^{12}\;A/m^{2}$. The results illustrate the frequency dependence of the domain-wall dynamics in the DMI-stabilized PMA nanowire. The simulations were performed at T = 0 K.

Figure 5a presents the early transient evolution of the local transverse magnetization component $m_{y}$ under different square-pulse frequencies at a fixed current density of $J=0.9\times 10^{12}\;A/m^{2}$. The results demonstrate pronounced frequency-dependent oscillatory dynamics of the DMI-stabilized Néel domain wall (DW) near the stepped pinning region. During the initial stage (t<2 ns), only weak oscillatory motion is observed for all frequencies, indicating that the injected spin-transfer torque initially produces limited DW excitation while energy gradually accumulates through repeated pulse cycles. Beyond approximately 2−2.5 ns, the oscillation amplitude increases significantly, revealing transient amplification and nonlinear excitation of the pinned DW. Higher frequencies, particularly 3−4 GHz, generate substantially larger oscillation amplitudes compared with lower frequencies, indicating a stronger dynamical response of the pinned DW to the periodic current excitation. Despite the enhanced oscillation amplitude, complete depinning is not observed at this subcritical current density, demonstrating that the stepped pinning barrier still dominates the long-term DW dynamics.

Figure 5b summarizes the oscillation amplitude as a function of pulse frequency at the higher current density of $J=1.3\times 10^{12}\;A/m^{2}$, corresponding to the near-threshold depinning regime. The oscillation amplitude exhibits a strongly non-monotonic frequency dependence characterized by several pronounced peaks near 1.0, 1.3, and 1.5 GHz**.** These frequencies correspond to the conditions where complete DW depinning was observed in the simulations, indicating that efficient synchronization between the square-pulse excitation and the localized oscillatory dynamics of the pinned DW enhances the transient oscillation response. In contrast, frequencies associated with lower oscillation amplitudes remain in the pinned oscillatory regime due to insufficient energy accumulation to overcome the stepped pinning barrier. The presence of distinct oscillation-amplitude maxima therefore supports frequency-selective resonance-assisted depinning of the DMI-stabilized DW under unipolar square-pulse excitation.

Overall, Figure 5 demonstrates two distinct dynamical regimes. At the lower current density, the pulse frequency primarily controls the strength of localized damped oscillation within the pinned regime, whereas near the critical current density, the pulse frequency becomes the determining factor governing nonlinear oscillation amplification and eventual depinning.

**Figure 6:**
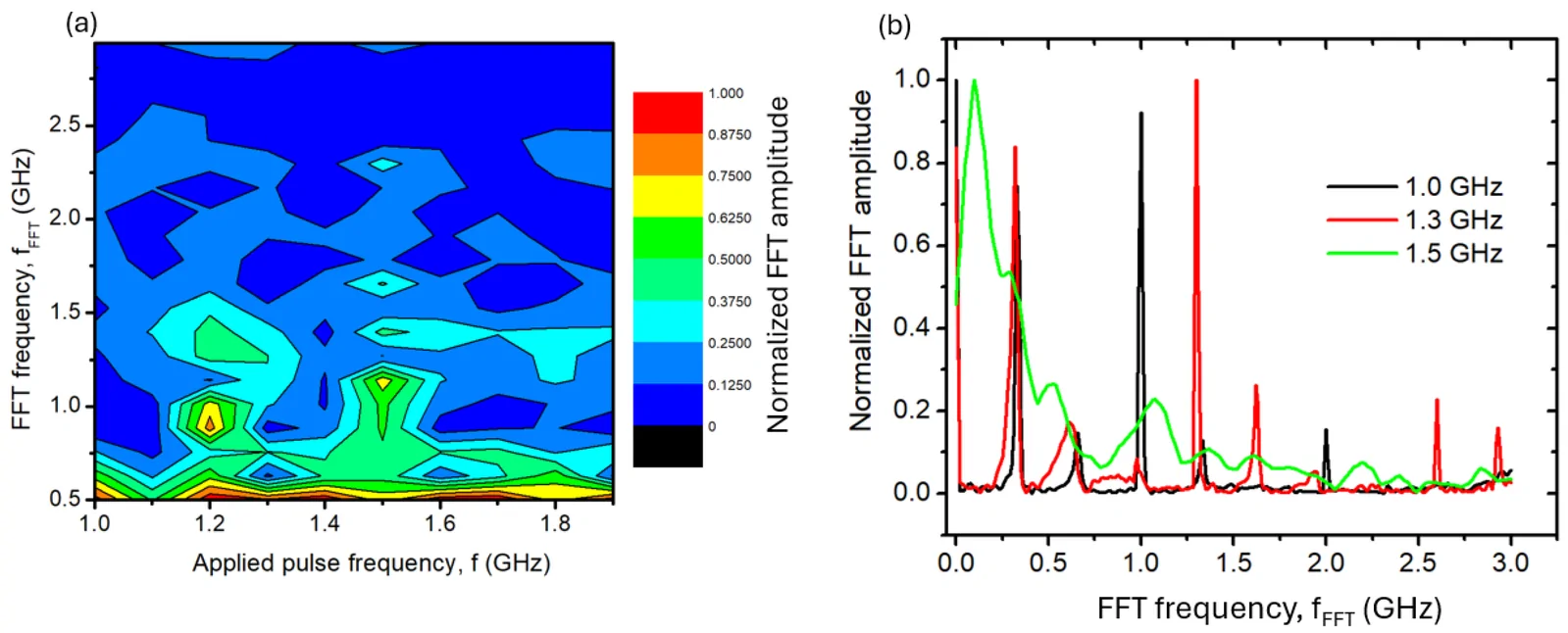
Frequency-dependent spectral response of the localized domain-wall dynamics at J = 1.3 × 10^12^ A m^−2^. (**a**) Normalized FFT-amplitude map as a function of applied pulse frequency and FFT frequency over the 1.0–1.9 GHz excitation range. (**b**) Representative normalized FFT spectra for applied pulse frequencies of 1.0, 1.3, and 1.5 GHz. The results reveal a frequency-selective spectral response of the pinned DW near the stepped region. The simulations were performed at T = 0 K.

Figure 6 presents the frequency-dependent spectral response of the local transverse magnetization measured near the stepped pinning region at J = 1.3 × 10^12^ A m^−2^. Figure 6a shows the normalized FFT-amplitude map as a function of the applied pulse frequency and FFT frequency over the excitation range from 1.0 to 1.9 GHz. The spectral response is clearly non-uniform across this range, showing that the DW does not respond equally to all excitation frequencies. Strong low-frequency components are present throughout the investigated range, while localized regions of enhanced spectral intensity appear at selected excitation frequencies. These features are consistent with the non-monotonic oscillation-amplitude behavior observed in Figure 5b and provide broader evidence of frequency-selective excitation of the pinned DW. Since the FFT amplitudes are normalized, the comparison mainly reflects the dominant spectral components and their positions rather than the absolute oscillation amplitude.

Figure 6b shows representative normalized FFT spectra for applied pulse frequencies of 1.0, 1.3, and 1.5 GHz. For the 1.0 GHz excitation, a pronounced spectral peak appears close to the applied frequency, indicating a strong periodic response of the localized DW dynamics. Similarly, for the 1.3 GHz case, a sharp spectral component is observed near the excitation frequency, consistent with the enhanced oscillatory response and depinning observed under this condition.

In contrast, the 1.5 GHz case exhibits a broader spectral response with pronounced low-frequency components and weaker features at higher frequencies. This broader spectrum indicates a more nonlinear and transient DW response involving deformation and displacement in addition to localized oscillation. Therefore, the response at 1.5 GHz cannot be described as a simple narrow forced oscillation but instead reflects more complex DW dynamics during the depinning process.

Overall, the broader FFT map together with the representative spectra shows that the DW response does not increase monotonically with pulse frequency. Instead, enhanced spectral responses occur within selected excitation-frequency ranges, indicating that the pulsed current couples more efficiently to the localized DW dynamics under particular conditions. The agreement between the frequency-dependent oscillation amplitudes in Figure 5b and the spectral features in Figure 6 supports the interpretation of frequency-selective, resonance-assisted depinning in the considered stepped geometry. These favorable frequencies are specific to the present pinning configuration and should not be interpreted as universal intrinsic resonance frequencies for arbitrary pinning sites.

More generally, the present results can be understood as an example of dynamically assisted escape from a metastable magnetic state. The stepped geometry creates a localized pinning potential that confines the DW in a metastable configuration, separated from propagation by an effective barrier. The pulsed Zhang–Li spin-transfer torque periodically excites the localized degrees of freedom of the pinned DW, and when this excitation couples efficiently to its characteristic dynamics, the resulting increase in oscillation amplitude and internal deformation facilitates barrier crossing and depinning [25,29,55].

This interpretation places the present mechanism within a broader class of processes in which a time-dependent external perturbation promotes escape from metastability [58]. A related example was reported by Abeed and Bandyopadhyay [59], where surface acoustic waves introduce time-dependent strain and modify the magnetic energy landscape of magnetostrictive nanomagnets through magnetoelastic coupling, enabling escape from metastable configurations. Related studies have also demonstrated surface-acoustic-wave-assisted DW pinning, depinning, and transport [60,61,62,63,64]. Although the microscopic coupling mechanisms differ, these systems illustrate the same general principle: an appropriately tailored time-dependent excitation can facilitate escape from a metastable state by modifying the effective barrier and/or exciting the relevant dynamical degrees of freedom. In this phenomenological sense, the process is analogous to simulated annealing; however, the present fixed-parameter T = 0 K dynamics are more precisely described as deterministic resonance-assisted dynamical barrier crossing. At finite temperature, the Brown thermal field provides an additional stochastic activation channel [42,56,58].

**Figure 7:**
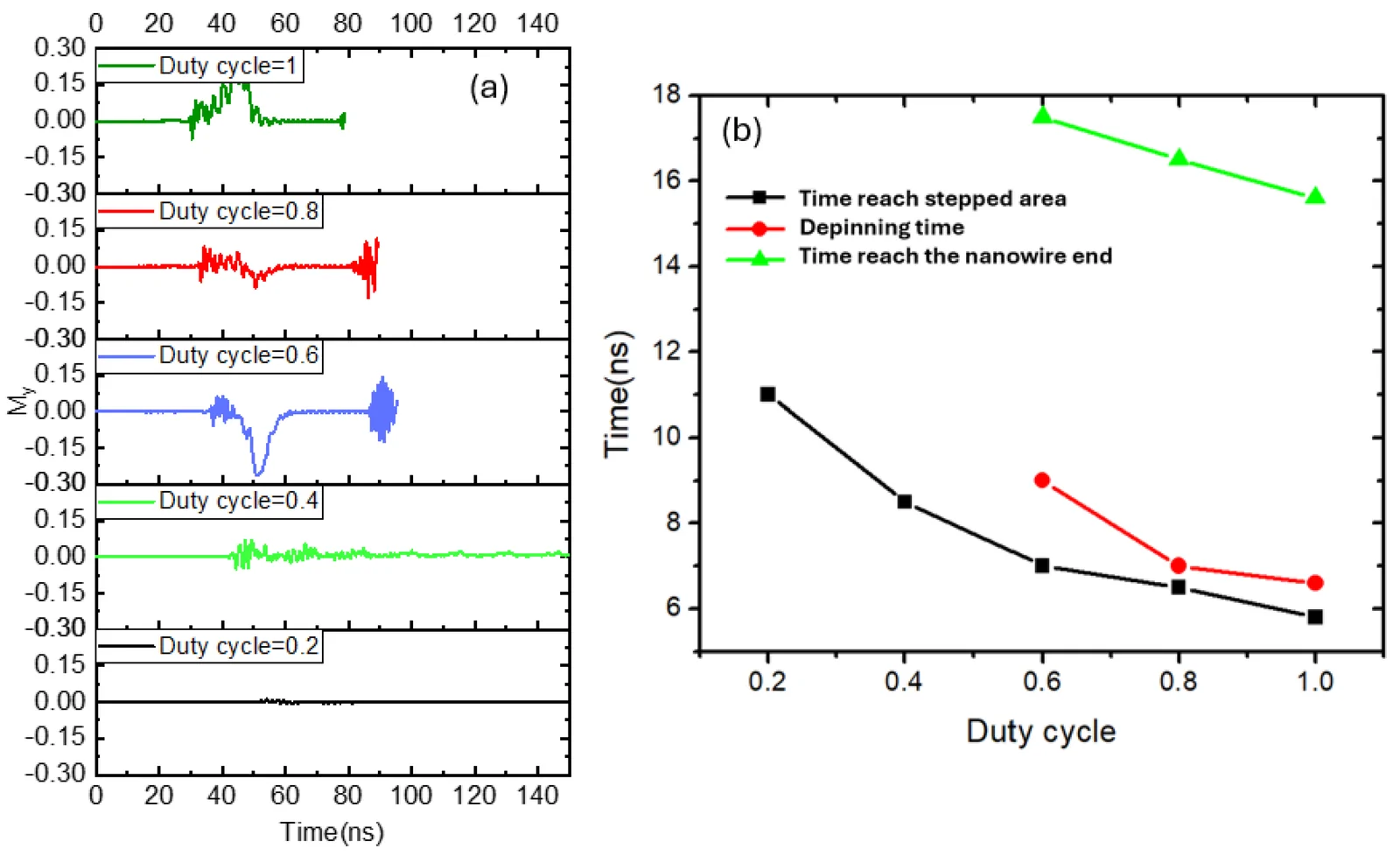
Duty-cycle-dependent dynamics of the DMI-stabilized Néel domain wall (DW) under unipolar square-pulse current excitation at $J=1.3\times 10^{12}\;A/m^{2}$ and f=1.3 GHz. (**a**) Temporal evolution of the local transverse magnetization component $m_{y}$ for different duty cycles. (**b**) Characteristic dynamical times as functions of duty cycle, including the arrival time at the stepped region, depinning time, and propagation time. The simulations were performed at T = 0 K.

Figure 7 presents the duty-cycle-dependent dynamics of the DMI-stabilized Néel domain wall (DW) in the stepped PMA nanowire under unipolar square-pulse current excitation with fixed current density $J=1.3\times 10^{12}\;A/m^{2}$ and pulse frequency f=1.3 GHz. The results demonstrate that the duty cycle strongly controls the nonlinear oscillatory behavior, transient energy accumulation, and depinning dynamics of the DW near the stepped pinning site.

Figure 7a shows the temporal evolution of $m_{y}$ for different duty cycles. At low duty cycles (DC=0.2 and 0.4), the DW exhibits weak damped oscillatory motion and remains pinned at the stepped region. In this regime, the short pulse ON duration and long OFF interval allow Gilbert damping to dissipate the injected energy before sufficient accumulation occurs for barrier crossing. Consequently, the oscillation amplitude remains below the critical depinning threshold.

As the duty cycle increases to 0.6, 0.8, and 1.0, the oscillatory response becomes significantly stronger and highly nonlinear. The larger ON duration enhances the effective spin-transfer torque excitation while reducing relaxation losses between successive pulses. This results in strong transient oscillation amplification and eventual depinning of the DW from the stepped barrier. The largest oscillation amplitude is observed for DC=1.0, corresponding to continuous excitation without OFF intervals, where energy accumulation becomes most efficient.

Figure 7b quantitatively summarizes the influence of duty cycle on the characteristic dynamical times. Increasing duty cycle systematically reduces the time required for the DW to reach the stepped region, the depinning time, and the propagation time toward the nanowire end. This indicates that larger duty cycles enhance both the effective DW velocity and the efficiency of energy transfer from the pulsed current excitation into the localized DW oscillation mode. The results further reveal the existence of a critical duty-cycle regime between 0.4 and 0.6, separating pinned oscillatory dynamics from complete depinning behavior.

Overall, the figure demonstrates that the duty cycle acts as a powerful dynamical control parameter governing nonlinear oscillation amplification, damping competition, transient energy accumulation, and resonance-assisted depinning in stepped PMA nanowires. These findings suggest that duty-cycle engineering provides an effective mechanism for controlling DW transport and depinning processes in future spintronic nanodevices.

**Figure 8:**
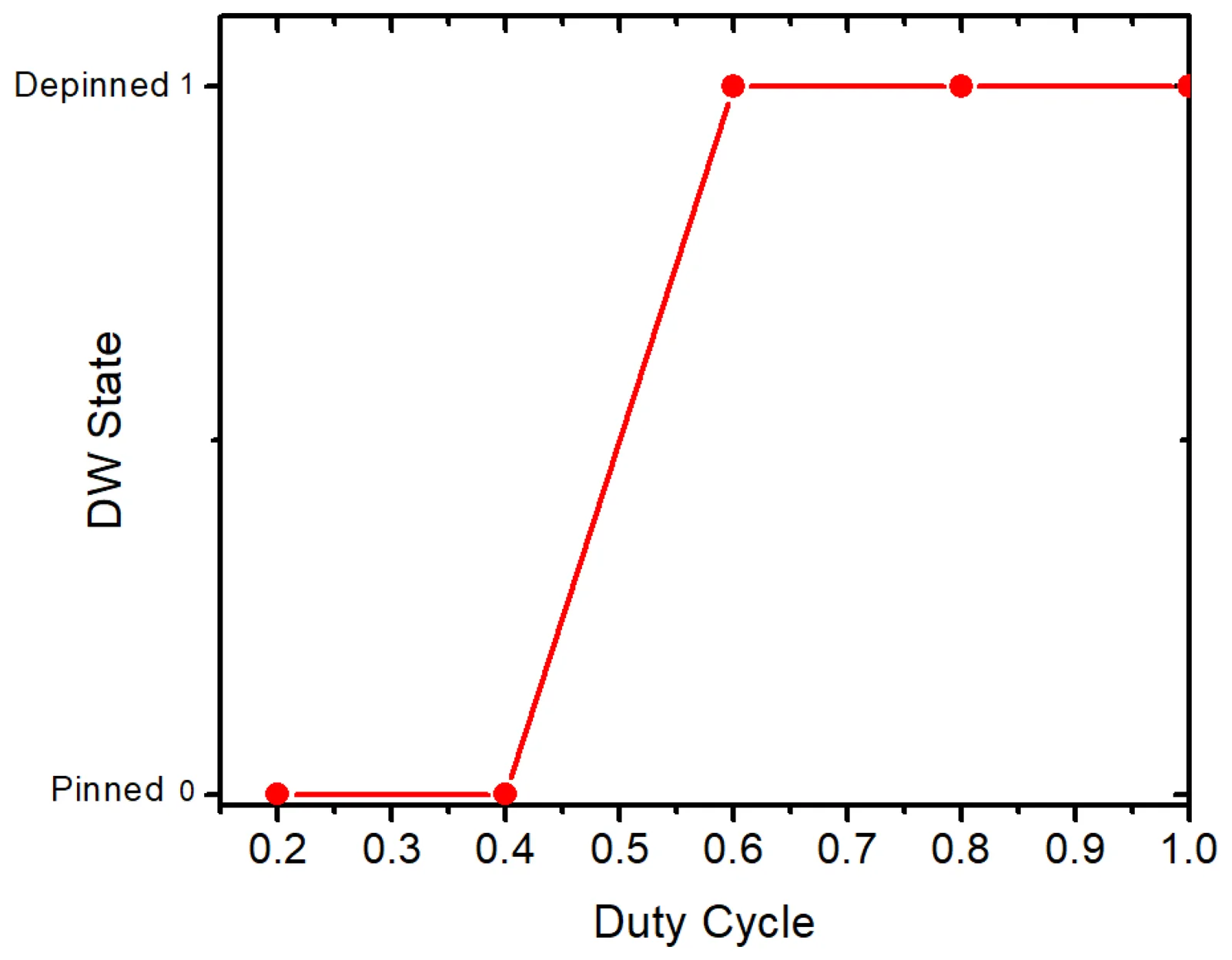
Duty-cycle-controlled dynamical phase diagram of the DMI-stabilized Néel domain wall (DW) under unipolar square-pulse current excitation at $J=1.3\times 10^{12}\;A/m^{2}$ and f=1.3 GHz. The diagram summarizes the pinned and depinned DW states as a function of duty cycle. The simulations were performed at T = 0 K.

Figure 8 presents the duty-cycle-controlled pinning–depinning transition of the DMI-stabilized Néel domain wall (DW) in the stepped PMA nanowire under unipolar square-pulse current excitation at fixed current density ($\;J=1.3\times 10^{12}\;A/m^{2}$) and pulse frequency (*f* = 1.3 GHz). The figure clearly demonstrates the existence of a critical duty-cycle threshold separating the pinned oscillatory regime from complete depinning behavior.

At low duty cycles (DC = 0.2 and 0.4), the DW remains trapped at the stepped pinning region despite undergoing transient oscillatory motion. In this regime, the short pulse ON duration combined with the relatively long OFF interval limits the net energy transferred into the DW oscillation mode. As a result, the injected spin-transfer torque excitation is continuously dissipated through Gilbert damping before sufficient energy accumulation can occur to overcome the pinning barrier.

A sharp dynamical transition occurs between duty cycles of 0.4 and 0.6, where the DW state abruptly changes from pinned to depinned. This behavior indicates the presence of a critical duty cycle required for resonance-assisted depinning. Above this threshold, the longer pulse ON duration significantly enhances transient energy accumulation while reducing relaxation losses between successive pulses. Consequently, the oscillation amplitude grows sufficiently to allow the DW to escape from the stepped pinning potential.

For higher duty cycles (0.6≤DC≤1.0), the DW consistently undergoes complete depinning, demonstrating that the system enters a stable nonlinear excitation regime where the effective spin-transfer torque continuously exceeds the combined effects of geometrical pinning and damping. The results therefore establish the duty cycle as a powerful dynamical control parameter governing the competition between energy injection and dissipation in pulsed-current-driven DW dynamics.

The observed duty-cycle-controlled phase transition further suggests that precise tuning of pulse ON/OFF ratios can provide an efficient mechanism for controlling DW transport, depinning probability, and nonlinear oscillatory behavior in stepped PMA spintronic nanodevices.

**Figure 9:**
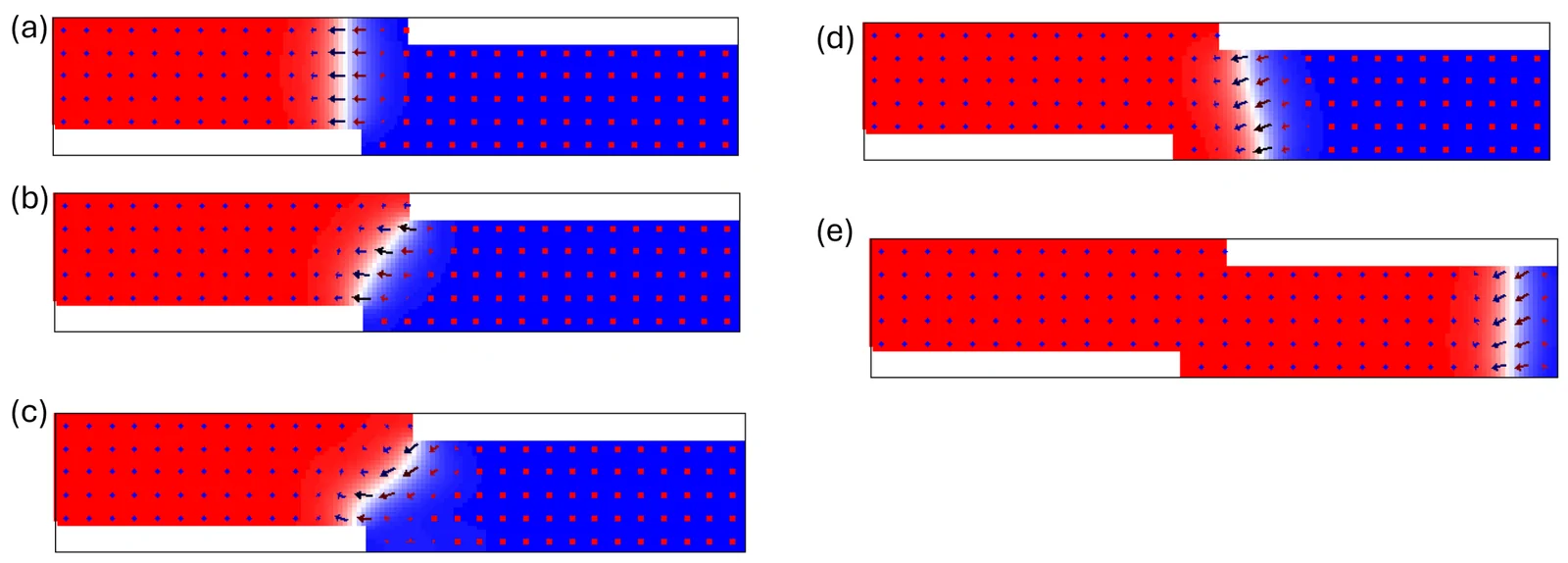
Representative micromagnetic snapshots illustrating the duty-cycle-dependent behavior of the DMI-stabilized Néel domain wall (DW) under unipolar square-pulse current excitation. Panels (**a**–**c**) show pinned DW configurations at lower duty cycles, while (**d**) and (**e**) correspond to the onset of depinning and the depinned state, respectively. The color map represents the normalized out-of-plane magnetization $m_{z}$, and the arrows indicate the local in-plane magnetization direction. The simulations were performed at T = 0 K.

Figure 9 presents representative micromagnetic snapshots illustrating the duty-cycle-dependent pinning and depinning behavior of the DMI-stabilized Néel domain wall (DW) in the stepped PMA nanowire under unipolar square-pulse current excitation. The figure demonstrates that the duty cycle not only controls whether the DW remains pinned or becomes depinned, but also strongly influences the spatial localization and internal deformation of the DW near the stepped pinning region.

Figure 9a–c correspond to the pinned regime observed at lower duty cycles. In Figure 9a, corresponding to DC = 0.2, the DW remains pinned outside the stepped trapping region. The DW is localized before reaching the geometrical confinement area, indicating that the injected pulse energy is insufficient to overcome the restoring interaction generated by the stepped barrier. In this regime, the long OFF interval between successive pulses allows strong damping relaxation, preventing effective energy accumulation within the DW oscillation mode.

In contrast, Figure 9b,c, corresponding to larger duty cycles, show that the DW becomes trapped inside the stepped region itself. Here, the DW penetrates deeper into the pinning potential and exhibits stronger deformation and localized oscillatory displacement near the geometrical discontinuity. The internal Néel spin structure becomes increasingly distorted, indicating enhanced transient excitation and stronger nonlinear oscillatory dynamics. These results suggest that increasing duty cycle promotes more efficient spin-transfer torque energy injection and reduces damping losses between successive pulses.

Figure 9d illustrates the onset of complete DW depinning for DC  =0.6. At this critical duty-cycle regime, the transient oscillation amplitude becomes sufficiently large to overcome the pinning barrier, allowing the DW to escape from the stepped region and propagate along the nanowire. The depinning process is accompanied by strong transient deformation and tilting of the DW structure, reflecting the nonlinear nature of the resonance-assisted depinning mechanism.

Finally, Figure 9e shows the DW after complete depinning and propagation away from the stepped region toward the nanowire end. The DW recovers a more stable Néel-like configuration after escaping the localized pinning potential, confirming successful pulse-driven transport through the stepped barrier.

Overall, Figure 9 provides direct micromagnetic evidence that the duty cycle governs the competition between damping relaxation, transient energy accumulation, and geometrical pinning. The results further confirm the existence of a critical duty-cycle threshold separating localized pinned oscillatory dynamics from complete resonance-assisted DW depinning.

**Figure 10:**
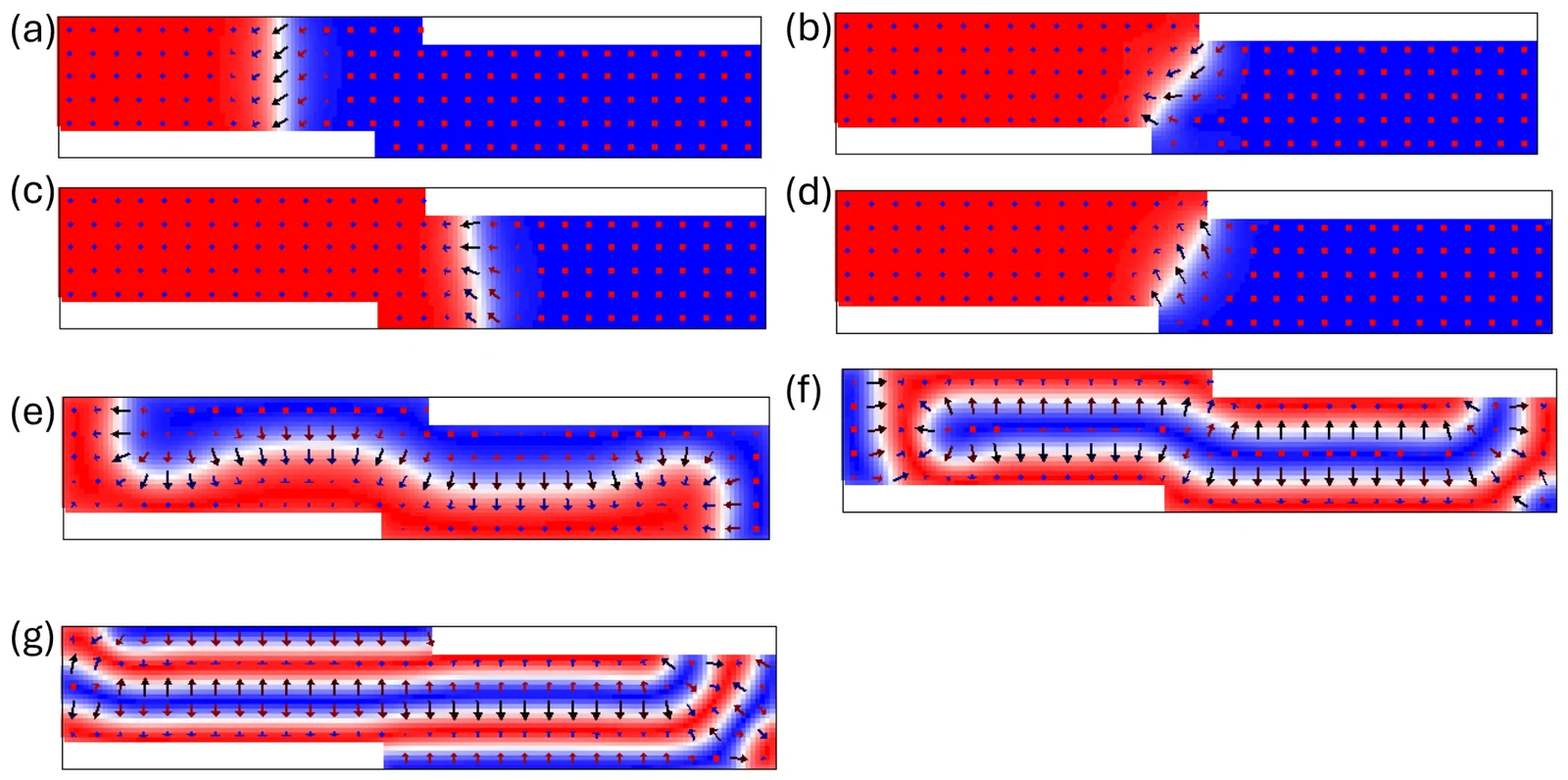
Influence of DMI strength on the DW configuration in the stepped PMA nanowire under continuous-current excitation at J = 1.3 × 10^12^ A m^−2^ (DC = 1). Panels (**a**–**c**) show successive stages of DW evolution for D=0.1  mJ/m^2^, while panels (**d**–**g**) correspond to D=0.5, 1.0, 1.5, and 2.0 mJ/m^2^, respectively. The color map represents the normalized out-of-plane magnetization $m_{z}$, and the arrows indicate the local in-plane magnetization direction. The simulations were performed at T = 0 K.

Figure 10 illustrates the influence of the Dzyaloshinskii–Moriya interaction (DMI) strength on the domain wall (DW) configuration and its interaction with the stepped pinning site under pulsed current excitation. A clear transition from conventional DW depinning to highly distorted chiral magnetic states is observed as the DMI constant increases.

Figure 10a–c represent successive snapshots of the same depinning event for D = 0.1 mJ/m^2^, illustrating the domain-wall evolution before, during, and after crossing the stepped pinning barrier. These snapshots provide a direct visualization of the depinning process and serve as a reference for understanding how increasing DMI strength modifies the domain-wall structure and its interaction with the stepped geometry.

For the lowest investigated DMI value, $D=0.1\;mJ/m^{2}$, shown in Figure 10a–c, the DW maintains a compact Néel-wall structure while interacting with the stepped region. The internal magnetization remains localized within a narrow wall width, and the wall successfully overcomes the geometrical pinning potential generated by the step. Consequently, the DW depins from the stepped area and propagates into the wider section of the nanowire. The coherent spin rotation and preserved wall profile indicate that weak DMI has only a minor influence on the internal DW structure, allowing efficient current-driven transport. The results demonstrate that the spin-transfer torque generated by the pulsed current is sufficient to overcome the step-induced energy barrier when the DMI strength is low.

Increasing the DMI strength to $D=0.5\;mJ/m^{2}$, as shown in Figure 10d, significantly alters the DW behavior. Although the wall reaches the stepped region, it remains trapped at the pinning site and fails to overcome the geometrical energy barrier. The stronger DMI stabilizes the chiral Néel configuration and enhances the interaction between the DW magnetization and the local magnetic environment near the step. Consequently, the effective pinning strength increases, preventing further DW propagation. This result indicates that a moderate DMI can suppress current-driven DW mobility by reinforcing geometrical pinning and stabilizing the wall structure at the defect location.

The enhanced pinning observed at D = 0.5 mJ/m^2^ suggests that moderate DMI increases the effective wall stiffness and chirality stabilization, thereby reducing the ability of the pulsed spin-transfer torque to deform the wall and overcome the geometrical barrier. This behavior highlights the competition between DMI-induced wall stabilization and current-driven depinning.

A further increase in DMI to $D=1\;mJ/m^{2}$, illustrated in Figure 10e, produces a substantial transformation of the DW structure. Instead of retaining a compact profile, the wall becomes highly elongated and distorted along the nanowire. The internal magnetization exhibits pronounced non-uniform rotation, leading to the formation of a curved and spatially extended magnetic texture. In this regime, the conventional rigid-wall approximation is no longer valid because the DMI energy becomes sufficiently strong to compete with exchange and anisotropy energies. As a result, the DW undergoes a shape transition from a localized wall into a nonlinear chiral configuration. The observed deformation suggests the onset of DMI-dominated dynamics, where the wall structure adapts to minimize the total magnetic energy rather than maintaining a rigid Néel-wall profile.

At $D=1.5\;mJ/m^{2}$, shown in Figure 10f, the DW evolves into a highly extended chiral state occupying a large portion of the nanowire. The magnetization continuously rotates along the wire length, indicating the emergence of a DMI-dominated magnetic texture. The concept of a single localized domain wall becomes less applicable because the magnetic structure is distributed over a much larger spatial region. The stepped area no longer acts as the primary factor controlling the magnetic behavior, while the DMI energy increasingly governs the overall magnetization configuration. This state represents a transition from localized DW dynamics to extended chiral magnetic ordering.

For the largest DMI value, $D=2\;mJ/m^{2}$, shown in Figure 10g, the chiral modulation becomes even more pronounced and extends almost throughout the entire nanowire. The magnetization exhibits a nearly continuous spin-spiral-like arrangement characterized by strong in-plane spin rotation and highly non-uniform magnetic ordering. In this regime, the magnetic configuration is dominated by DMI rather than by geometrical pinning, resulting in the formation of an extended chiral texture instead of a localized DW. The results indicate that excessively large DMI strengths fundamentally alter the nature of the magnetic state and suppress conventional DW pinning–depinning dynamics.

Overall, Figure 10 reveals a DMI-induced transition from efficient DW depinning at low DMI, to enhanced pinning at intermediate DMI, followed by severe wall deformation and the eventual formation of extended chiral magnetic states at high DMI. These findings demonstrate that DMI plays a crucial role in controlling both the internal structure and transport properties of DWs in stepped PMA nanowires, providing an effective mechanism for tuning current-driven magnetic dynamics and chirality-dependent magnetic states in future spintronic devices.

**Figure 11:**
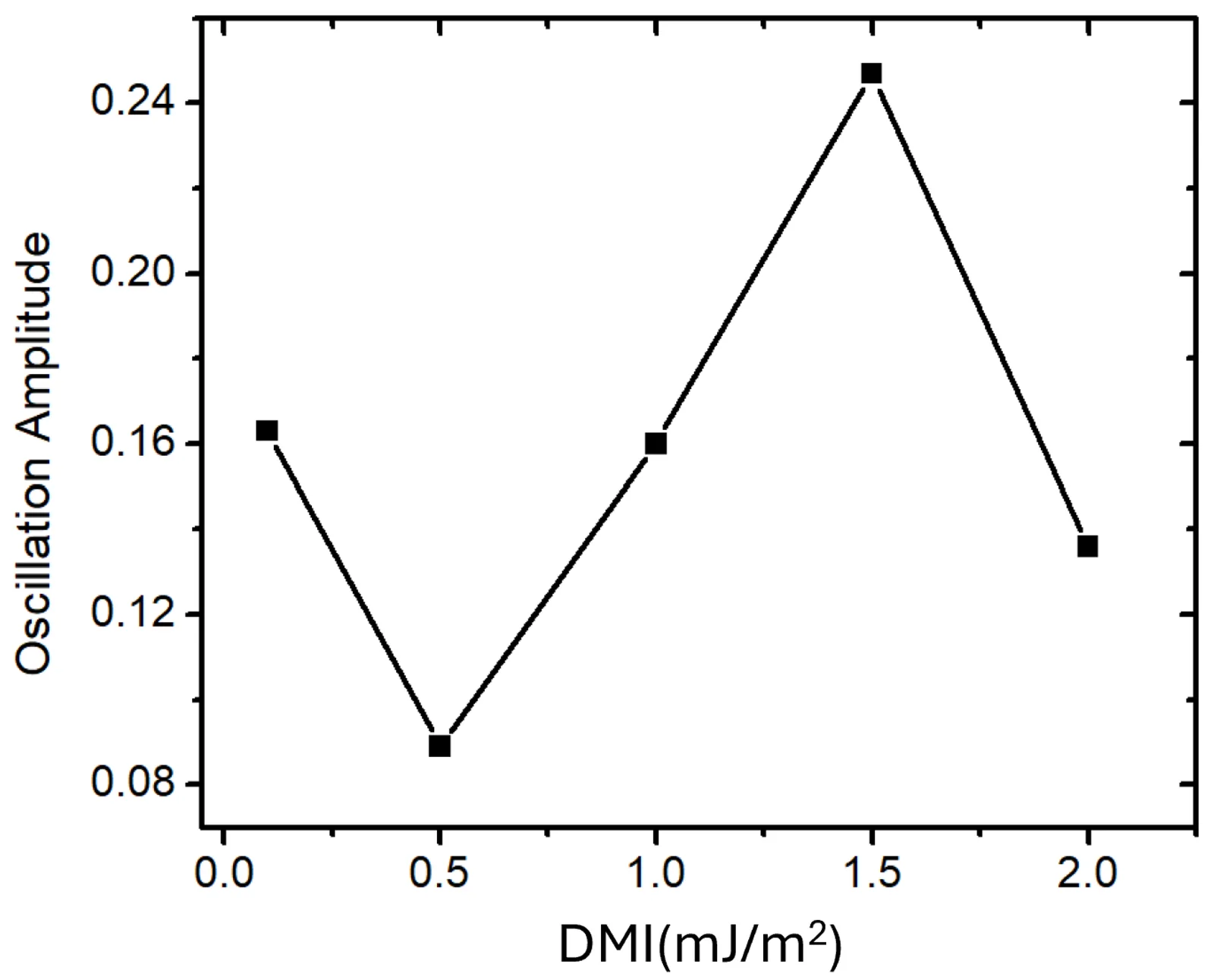
Oscillation amplitude A_osc as a function of DMI strength under continuous-current excitation at J = 1.3 × 10^12^ A m^−2^ (DC = 1). The simulations were performed at T = 0 K.

**Figure 12:**
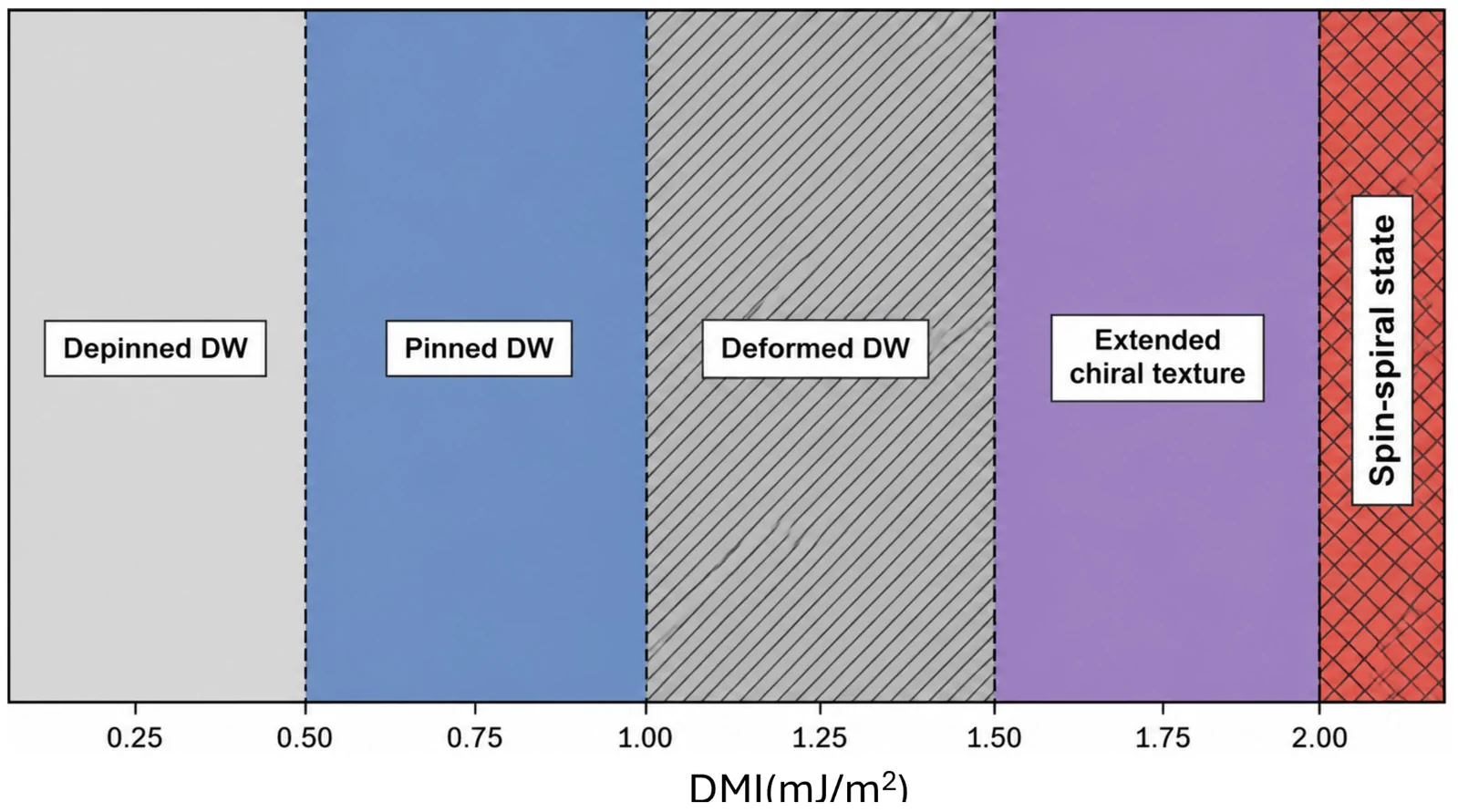
DMI-dependent dynamical phase diagram showing the evolution of domain-wall states with increasing DMI strength in the stepped PMA nanowire. The simulations were performed at T = 0 K.

Figure 11 illustrates the influence of DMI strength on the DW oscillation amplitude under continuous-current excitation at J = 1.3 × 10^12^ A m^−2^ (DC = 1). Figure 11 presents the DMI dependence of the domain-wall oscillation amplitude, while the corresponding DMI-dependent dynamical phase diagram is shown in Figure 12. Figure 11 shows that the oscillation amplitude exhibits a pronounced non-monotonic dependence on the DMI strength. At $D=0.1\;mJ/m^{2}$, the oscillation amplitude is relatively large, indicating that the DW remains sufficiently flexible to respond to the pulsed current excitation and overcome the geometrical barrier. Increasing the DMI to $0.5\;mJ/m^{2}$ significantly reduces the oscillation amplitude, corresponding to a more stable and strongly pinned DW configuration at the stepped region. This reduction suggests that moderate DMI increases the effective rigidity of the DW and suppresses its dynamic response.

As the DMI is further increased to $1.0\;mJ/m^{2}$, the oscillation amplitude rises again, coinciding with a noticeable deformation of the DW structure. At this stage, the DMI-induced chiral torque becomes sufficiently strong to modify the internal spin arrangement within the wall, producing enhanced internal excitations and larger oscillatory motion. The maximum oscillation amplitude occurs at $D=1.5\;mJ/m^{2}$, indicating the existence of an optimal DMI range where the energy supplied by the pulsed current couples most efficiently to the DW internal modes. In this regime, the DW evolves into an extended chiral magnetic texture that can support large-amplitude nonlinear oscillations.

For $D=2.0\;mJ/m^{2}$, the oscillation amplitude decreases despite the stronger DMI. This behavior indicates that excessively large DMI stabilizes highly ordered chiral configurations resembling spin-spiral states. Such configurations become energetically rigid and therefore less responsive to external excitation, limiting the growth of oscillatory motion.

To further clarify the influence of DMI on domain-wall behavior, Figure 12 summarizes the DMI-dependent dynamical phase evolution observed in the simulations. Increasing DMI drives the system through a sequence of distinct dynamical regimes: depinned DW motion, pinned DW state, deformed DW configuration, extended chiral texture, and finally a spin-spiral-like magnetic state. This progression demonstrates that DMI not only influences the depinning efficiency but fundamentally changes the magnetic configuration itself. The results therefore reveal a DMI-induced dynamical phase transition in stepped PMA nanowires, where the competition between exchange interaction, perpendicular magnetic anisotropy, geometrical pinning, and chiral DMI energy governs the stability and dynamics of the DW. This DMI-driven transition from conventional depinned domain-wall motion to extended chiral textures and spin-spiral-like states highlights the important role of DMI in controlling both resonance-assisted dynamics and magnetic-state evolution in the considered stepped PMA nanowire.

Overall, Figure 11 identifies an optimal DMI regime near D ≈ 1.5 mJ/m^2^, where the domain wall (DW) exhibits the largest oscillation amplitude and the strongest dynamic response under pulsed-current excitation. This result indicates that the coupling between the external pulse excitation and the intrinsic DW oscillation modes is maximized at intermediate DMI strengths. Consequently, careful DMI engineering may provide an approach for tuning resonance-assisted energy transfer and DW dynamics in the considered system. The non-monotonic dependence of oscillation amplitude on DMI reveals a competition between chiral stabilization and domain-wall flexibility. At low DMI values, the DW remains relatively mobile but exhibits limited oscillatory excitation. As the DMI increases, stronger chiral interactions enhance internal DW excitations and promote more efficient energy transfer from the pulsed current into the DW oscillation modes, resulting in a maximum oscillation amplitude near D = 1.5 mJ/m^2^. However, further increases in DMI drive the system toward extended chiral textures and spin-spiral-like states, as summarized in Figure 12, reducing the ability of the magnetic structure to sustain coherent oscillations. These results demonstrate the existence of an optimal DMI window for maximizing resonance-assisted DW dynamics.

**Figure 13:**
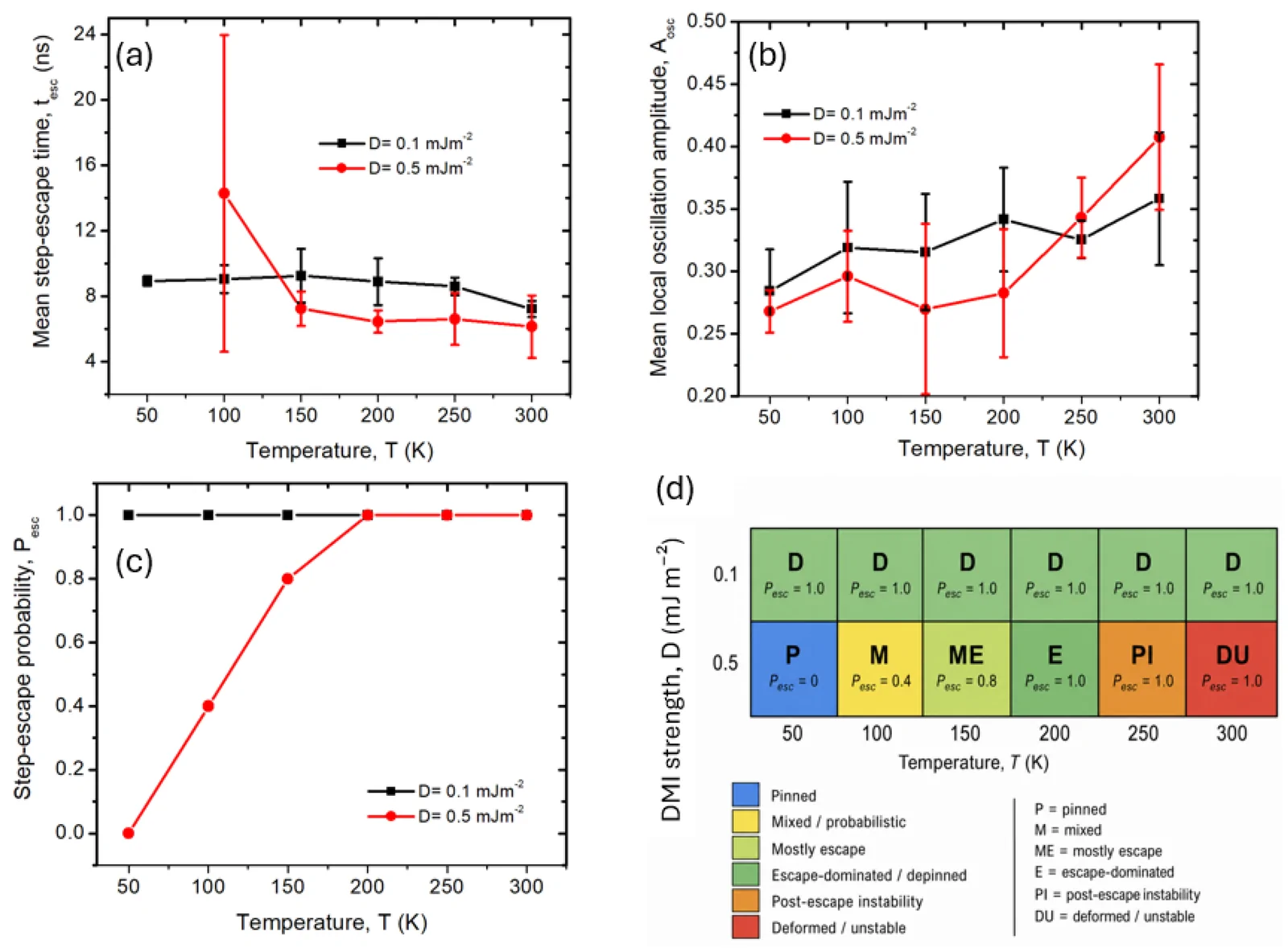
Statistical analysis of the temperature-dependent domain-wall dynamics for D = 0.1 and 0.5 mJ m^−2^ under continuous-current excitation at J = 1.3 × 10^12^ A m^−2^ (DC = 1). Five independent thermal-noise realizations were used at each temperature. (**a**) Mean step-escape time with standard-deviation error bars, (**b**) mean local oscillation amplitude A_osc_ with standard deviation, (**c**) step-escape probability P_esc_, and (**d**) dynamical phase diagram showing pinned, probabilistic escape, depinned, post-escape unstable, and thermally deformed regimes.

Figure 13 summarizes the effect of temperature on the domain-wall dynamics for D = 0.1 and 0.5 mJ m^−2^ under continuous-current excitation. Five independent thermal-noise realizations were performed at each temperature. For each realization, the simulation protocol consisted of 2 ns of zero-current thermal equilibration, followed by up to 100 ns of continuous-current excitation and 10 ns of post-current relaxation, giving a maximum nominal simulation duration of 112 ns. As shown in Figure 13a, the case with D = 0.1 mJ m^−2^ shows relatively stable step-escape times over the investigated temperature range. The mean escape time stays close to 9 ns at low and intermediate temperatures and decreases gradually to about 7.2 ns at 300 K. This indicates that the DW can overcome the stepped pinning region relatively easily when the DMI strength is weak.

A different behavior is observed for D = 0.5 mJ m^−2^. At 50 K, none of the five trajectories escape from the stepped region. At 100 K, only two out of five trajectories escape, with a mean escape time of about 14.3 ns for the successful runs and a relatively large standard deviation. This variation reflects the stochastic nature of the DW motion near the transition from pinning to depinning. At 150 K, four out of five trajectories escape, and the mean escape time of the successful trajectories decreases to about 7.2 ns. For T ≥ 200 K, all five trajectories cross the stepped region, with mean escape times remaining around 6–7 ns. These results show that thermal fluctuations assist the DW in overcoming the stronger pinning associated with the higher DMI value.

The local oscillation amplitudes in Figure 13b show a similar temperature-dependent behavior. For D = 0.1 mJ m^−2^, A_osc_ changes only slightly with temperature, indicating that the local oscillatory motion of the DW remains relatively stable. For D = 0.5 mJ m^−2^, however, the oscillation amplitude becomes more sensitive to temperature and increases at higher temperatures, reaching its largest value near 300 K. The standard-deviation error bars also become more noticeable at several temperatures, reflecting the differences between independent thermal trajectories.

The step-escape probability shown in Figure 13c provides a direct view of this transition. For D = 0.1 mJ m^−2^, P_esc_ = 1.0 at all investigated temperatures, confirming reliable step escape. In contrast, for D = 0.5 mJ m^−2^, P_esc_ increases from 0 at 50 K to 0.4 at 100 K and 0.8 at 150 K, before reaching 1.0 for T ≥ 200 K. This gradual increase shows that temperature not only affects the oscillatory response of the DW but also changes the likelihood that it can overcome the stepped pinning barrier. The intermediate probabilities at 100 and 150 K also demonstrate the importance of using multiple thermal trajectories to describe this transition.

The main dynamical regimes are summarized in the phase diagram in Figure 13d. For D = 0.1 mJ m^−2^, the DW remains in the depinned regime over the entire temperature range. For D = 0.5 mJ m^−2^, the behavior changes progressively with temperature. The DW is pinned at 50 K, enters a mixed probabilistic regime at 100 K, and becomes mostly depinned at 150 K. At 200 K, all five trajectories escape from the stepped region, giving an escape-dominated regime. At 250 K, all trajectories also escape, although some later return toward the stepped region or become re-pinned, indicating post-escape instability. At 300 K, the DW still crosses the step, but stronger thermal fluctuations lead to noticeable deformation and an unstable magnetic state.

Overall, the results show that the influence of temperature depends strongly on the DMI strength. For D = 0.1 mJ m^−2^, depinning remains robust across the full temperature range, whereas D = 0.5 mJ m^−2^ shows a clear evolution from pinning to probabilistic escape and finally to thermally disturbed motion. Under the present simulation conditions, the intermediate-temperature region around 200 K provides the best balance between overcoming the pinning barrier and maintaining stable DW transport. The present thermal analysis is limited to continuous-current excitation and two representative DMI strengths. A broader study combining temperature with pulse frequency and duty cycle would be a useful direction for future work.

These results demonstrate that thermal activation does not simply promote depinning; instead, its effect depends strongly on the underlying DMI-stabilized magnetic configuration and may either facilitate depinning or induce deformation of the magnetic texture. Thermal fluctuations effectively reduce the energy barrier associated with the stepped pinning site, enabling depinning even when DMI-enhanced chirality stabilization suppresses motion at lower temperatures. This behavior suggests that thermal fluctuations not only assist depinning but may also destabilize coherent domain-wall propagation, resulting in a thermally induced deformed magnetic state. This phase transition demonstrates that temperature can fundamentally alter the balance between thermal activation, DMI-induced chirality stabilization, and geometrical pinning, thereby governing the transition between pinned, depinned, and partially deformed domain-wall states.

## 4. Conclusions

In this work, the dynamics of DMI-stabilized Néel domain walls in a stepped perpendicularly magnetized nanowire driven by unipolar square pulsed currents were systematically investigated using micromagnetic simulations. The influence of current density, pulse frequency, duty cycle, Dzyaloshinskii–Moriya interaction (DMI), and temperature on domain-wall (DW) pinning and depinning behavior was analyzed.

The results revealed that the applied current density strongly influences the DW dynamic response. At low current densities, the DW remained pinned at the stepped region and exhibited weak oscillatory motion. Increasing the current density enhanced the oscillation amplitude and produced transitions from weak pinned oscillations to nonlinear oscillatory states. Complete depinning occurred when the current density exceeded a critical value, demonstrating the existence of distinct dynamical regimes that can be controlled through current-induced spin-transfer torque.

The duty cycle was found to play a crucial role in determining the DW state. Low duty cycles provided insufficient energy to overcome the geometrical pinning potential, resulting in pinned configurations. Increasing the duty cycle increased the effective torque acting on the DW and promoted depinning. A critical duty-cycle threshold was identified, above which stable depinning was achieved. These findings demonstrate that duty-cycle engineering provides an effective approach for controlling DW motion without increasing the peak current density.

The DMI strength significantly modified both the static and dynamic properties of the DW. For weak DMI values, the DW maintained a conventional Néel-wall configuration and readily depinned from the stepped region. An intermediate DMI strength produced enhanced pinning and reduced oscillation amplitudes, indicating stronger confinement at the geometrical defect. Further increases in DMI generated substantial deformation of the DW structure, leading to extended chiral textures and spin-spiral-like magnetic states. The oscillation amplitude exhibited a non-monotonic dependence on DMI, reaching a maximum near D = 1.5 mJ/m^2^, suggesting the existence of an optimal DMI regime for resonance-assisted DW excitation. The maximum oscillation amplitude observed near D = 1.5 mJ/m^2^ indicates the existence of an optimal balance between chiral stabilization and domain-wall flexibility for enhanced dynamical excitation by the pulsed current.

Thermal effects were found to depend strongly on the DMI strength. For D = 0.1 mJ/m^2^, the DW remained relatively stable over the investigated temperature range, exhibiting only moderate changes in oscillation amplitude and depinning behavior. In contrast, for D = 0.5 mJ/m^2^, the temperature significantly altered the pinning characteristics, resulting in transitions between pinned, depinned, and partially deformed states. The present thermal analysis was restricted to continuous-current excitation and two representative DMI strengths; a systematic investigation of the combined effects of temperature, pulse frequency, and duty cycle remains beyond the scope of this work.

Dynamical phase diagrams constructed as functions of current density, duty cycle, DMI strength, and temperature revealed clear transitions among pinned oscillation, nonlinear oscillation, deformed DW states, extended chiral textures, spin-spiral-like configurations, and complete depinning. The results demonstrate that resonance-assisted DW dynamics can be effectively controlled through pulse engineering and DMI tuning.

A key outcome of this work is the demonstration that resonance-assisted depinning can be achieved through pulse engineering without increasing the peak current density. By appropriately tuning the pulse frequency and duty cycle, the domain wall develops enhanced oscillatory motion over successive pulse cycles, facilitating the crossing of the geometrical pinning barrier. This mechanism provides an effective approach for controlling chiral domain-wall motion and demonstrates the potential of resonance-based operation for spintronic memory and logic applications. Overall, this study provides a systematic characterization of the effects of pulsed-current excitation, DMI, and thermal fluctuations on domain-wall dynamics in stepped PMA nanowires. The results show that current density, pulse frequency, duty cycle, and DMI strength strongly influence the dynamical regimes leading to resonance-assisted depinning and magnetic-state evolution, while thermal fluctuations modify the stability of these states. These findings provide insight into controlling chiral domain-wall motion through pulse engineering and DMI tuning and demonstrate the potential of resonance-assisted excitation for spintronic applications.

## Figures and Tables

**Table 1:** Geometrical dimensions, magnetic properties, and simulation parameters used for investigating pulsed-current-driven domain wall pinning and depinning in stepped DMI-stabilized PMA nanowires.

Parameter	Symbol	Value
Nanowire length	L	300 nm
Nanowire width	W	80 nm
Nanowire thickness	t	3 nm
Step width	λ	30 nm
Step depth	d	15 nm
Saturation magnetization	Ms	800 kA/m
Exchange stiffness	A	1.5 × 10^−11^ J/m
Perpendicular anisotropy constant	Ku	0.9 × 10^6^ J/m^3^
Gilbert damping constant	α	0.01
Spin polarization	P	0.4
Non-adiabaticity parameter	β	0.1
DMI constant	D	0.1–2.0 mJ/m^2^
Current density	J	0.6–1.3 × 10^12^ A/m^2^
Pulse frequency	f	0.5–4.0 GHz
Duty cycle	DC	0.2–1.0
Temperature	T	50–300 K
Cell size	Δx × Δy × Δz	2 × 2 × 1 nm^3^
Simulation package	–	MuMax3
Domain wall type	–	Néel DW

## Data Availability

Data is contained within the article.
